# Precision Medicine for Breast Cancer: The Paths to Truly Individualized Diagnosis and Treatment

**DOI:** 10.1155/2018/4809183

**Published:** 2018-05-09

**Authors:** Eleanor E. R. Harris

**Affiliations:** Department of Radiation Oncology, Case Western Reserve University and University Hospitals, Cleveland, OH, USA

## Abstract

Precision medicine in oncology seeks to individualize each patient's treatment regimen based on an accurate assessment of the risk of recurrence or progression of that person's cancer. Precision will be achieved at each phase of care, from detection to diagnosis to surgery, systemic therapy, and radiation therapy, to survivorship and follow-up care. The precision arises from detailed knowledge of the inherent biological propensities of each tumor, rather than generalizing treatment approaches based on phenotypic, or even genotypic, categories. Extensive research is being conducted in multiple disciplines, including radiology, pathology, molecular biology, and surgical, medical, and radiation oncology. Clinical trial design is adapting to the new paradigms and moving away from grouping heterogeneous patient populations into limited treatment comparison arms. This review touches on several areas invested in clinical research. This special issue highlights the specific work of a number of groups working on precision medicine for breast cancer.

## 1. Background

The National Research Council released a consensus study report in 2011 entitled “Toward Precision Medicine” [1]. The report proposes to define a “new taxonomy” of disease based on molecular and environmental determinants rather than signs and symptoms, harnessing the power of big data networks and expanding information about the molecular determinants of disease to first define these molecular subtypes and then apply that knowledge to personalized treatment approaches based on the individual patient's precise molecular pathology. The imprecise approach of “one size fits all” treatments to patients with general classes of disease, such as breast cancer, has been undergoing a paradigm shift for several years, with the identification of molecular pathways predicting both tumor biology as well as response to therapy. But the holy grail of truly individualized treatment based on each patient's specific tumor molecular and environmental subtype and application of a specific effective treatment regimen for that cancer subtype is the subject of ongoing research on many fronts. The “New Taxonomy” proposes to develop a more accurate disease classification system based on molecular biology [2]. A “Knowledge Network” will be created to incorporate genomic data from a large variety of sources, including DNA sequencing and other molecular technologies, basic science research, clinical trial data, observational studies, and electronic health records to analyze connections between the different sets of information to define disease classifications and to test potential targeted treatments. In January 2015, the Obama administration in the United States launched the Precision Medicine Initiative [3], specifically to improve the treatment and cure of diseases like cancer, which is the near-term disease focus of the initiative. This cancer focused component is designed to address existing challenges to achieving precision cures, including drug resistance, tumor genomic heterogeneity, reliable markers of tumor response, and optimal methods for combining multiple agents or modalities most effectively. Large scale data collection for over a million people will be collated, pilot studies of treatments and longitudinal studies of outcomes conducted. This massive federally sponsored initiative promises to accelerate precision medicine advancements more comprehensively, as data will be openly shared with investigators, physicians, and the public.

Precision medicine encompasses a very broad spectrum of clinical and basic science disciplines. True personalization of treatment will account for the individual patient's genetics and genetic predispositions, the composition of their breast tissue, the “omic” profile of their cancer and consequent biologic propensities, tissue microenvironment, comorbid conditions, lifestyle, patient preference, and quality of life. Precision medicine enters the scene even before the cancer diagnosis, in the arenas of prevention and detection. After diagnosis, precision medicine requires a fundamental shift in the traditional approaches to clinical trial design, as ever smaller bins of molecularly staged patients receiving novel targeted agents will not provide the statistical power to detect significance for outcome endpoints such as local control or overall survival under traditional definitions.

Breast research cancer is well poised to make substantive advances in the precision medicine era. Much ground work has been laid. Some of the work being done to reach the goals of precision medicine in breast cancer detection, prevention, diagnosis, and treatment are highlighted throughout this special issue.

## 2. Precision Medicine Disciplines in Breast Cancer

### 2.1. Screening Diagnostics

Traditional screening guidelines have defined parameters such as age, risk level defined by mammogram appearance and breast biopsy findings, and genetic predisposition to guide the use of imaging studies (mammogram and ultrasound primarily) for population based screening. This approach is imprecise as it leads to overscreening of some and underscreening of other segments of the population. A precision medicine approach supports gene sequencing for profiling of individual genetic risk and determining the screening methodology and frequency based on individual risk level. An individualized risk score is based on factors including genetics, body mass index, family history, and imaging features such as breast density. This approach could allow less frequent use of unnecessary tests leading to overdiagnosis and overtreatment. It could also lessen underscreening, particularly in the younger population where early detection is more challenging yet arguably more impactful to outcomes. Low risk patients could have screening less often or not at all, saving cost and unnecessary tests due to false positive results. Very high risk patients could begin screening younger, have it performed more often, or include functional imaging such as MRI. Risk-based screening is being studied in clinical trials underway [4].

In the general screening population, revisions of risk stratification models are being adopted. Breast density has emerged as a significant risk factor for cancer incidence. The breast imaging and reporting data system (BIRADS) incorporates four categories of breast density to be included in the interpretation of screening mammograms: fatty; scattered fibroglandular densities; heterogeneously dense; and extremely dense [5]. In a large Swedish mammographic screening study of over 15,600 women ages 45 to 59 followed for 25 years, dense breast tissue was associated with a 1.57 adjusted relative risk of cancer incidence and 1.9 relative risk of breast cancer mortality [6]. The Breast Cancer Surveillance Consortium conducted a case control study from the SEER database and prospective risk factor collection from 1996 to 2012 from their registry of breast imaging facilities, reporting the BI-RADs breast density, among other risk factors [7]. Using an outcome of population attributable risk proportion of developing breast cancer, breast density was the most prevalent factor for all age groups. Body mass index was also highly contributory. Such data have led to studies assessing the timing, frequency, and imaging modalities optimally used for effective screening of women with dense breast tissue, potentially including functional imaging such as MRI. The Breast Cancer Surveillance Consortium is the first national organization to formally incorporate breast density into its risk calculation algorithm [8]. Interventions to reduce breast density using diet, dietary supplements, exercise, and pharmacologic agents have been investigated [9, 10]. The impact of lifestyle interventions such as healthy eating, weight reduction, and physical activity in reducing the incidence of breast cancer could be substantial.

### 2.2. Molecular Subtype and Systemic Therapy

Molecular subtypes of breast cancer defining phenotypic behaviors based on molecular determinants have been identified [11], although there is significant heterogeneity within and between these subtypes, which require further refinement [12]. Molecular subtyping using available immunohistochemical assays is the current standard for diagnostic characterization of all breast cancer patients and an integral component of treatment decision making. Perou et al. identified “molecular portraits” from 65 breast cancer specimens using DNA microarray technique for quantitative analysis of gene expression patterns in over 8000 genes [11]. These patterns were grouped into subtypes based on the differences between the expression profiles. Based on this seminal work and other studies, the most common general molecular subtypes characterized currently include the following classifications: Luminal A (estrogen receptor (ER) positive, Her2 negative AND Ki-67 low < 14%, OR Ki-67 intermediate 14–19%, and progesterone receptor (PG) high > 20%); Luminal B (ER positive, Her2 negative, AND Ki-67 intermediate 14–19%, and PG low/negative OR Ki-67 high > 20%, OR Her2+) [13]; Her2 enriched (ER negative, PR negative, and Her2 positive); and triple negative/basal-like (ER negative, PR negative, and Her2 negative). The use of these immunohistochemical assays to identify the expression profile (with FISH testing in equivocal Her2 cases) is the simplest and most widely available system. The molecular subtypes translate into phenotypes which correlate with tumor behavior, survival outcomes, and response to treatment. Overall, there is a worse prognosis for the triple negative and Her2+ groups, as well as a clear distinction between two ER+ groups [14]. Correlation between molecular subtype and local-regional recurrence risk has also been observed [15]. A meta-analysis of published breast cancer gene expression profiles and associated clinical data collated the various signatures into three main biologic pathways: proliferation; estrogen receptor (ER) expression, and Her2 expression [16]. The four basic intrinsic subtypes originally identified by Perou were validated, and proliferation pathways were noted to be the most highly prognostic. These investigators conclude that different molecular signatures actually provide similar prognostic information due to identifying common pathways.

Personalization of breast cancer therapy over the past two decades has relied primarily on these subtypes. However, further refinement of these classifications is the focus of current basic science and clinical research, to achieve better outcomes in patients along the spectrum of risk within each broad subtype. A variety of multigene assays are in clinical use or under investigation, which further define the molecular characteristics of the cancers' dominant biologic pathways. These gene arrays are most commonly used to inform the decision making regarding systemic therapy and are being investigated for other prognostic uses, such as predicting locoregional recurrence to inform patient selection for surgery or radiation treatment. One of the first multigene assays was reported by Paik et al., using RT-PCR profiles of 21 preselected genes in a group of ER positive, node negative patients previously treated with tamoxifen alone [17]. This 21-gene assay categorized patients into groups of low, intermediate, or high risk for distant recurrence and resulted in the development of a predicative scoring system known commercially as Oncotype DX Recurrence Score (Genomic Health). This gene assay is commonly used in clinical practice to guide recommendations regarding the potential efficacy of systemic chemotherapy in addition to endocrine therapy in lower risk early stage ER positive patients. The 21-gene assay has also been shown to predict the risk of local-regional recurrence, suggesting a role in patient selection for adjuvant radiotherapy [18]. A 70-gene array of mRNA expression (commercially known as Mammaprint [19], Agendia) is also tested on tumor tissue, and its gene panel includes pathways of growth signaling, apoptosis, replication, metastasis, and angiogenesis [20]. The assay provides a good or poor prognostic signature that discriminates between risk of distant metastasis among a larger group of breast cancers, including those with Her2-positive and node positive cancers. It is similarly used clinically to guide use of systemic chemotherapy or sometimes to provide further prognostic information in women with intermediate recurrence scores after the 21-gene assay has been performed. A 50-gene mRNA expression array called Predication Analysis of Microarrays (PAM50, Prosigna, Nanostring Technologies) was designed to classify intrinsic molecular subtype [21]. The assay provides a prognostic score called risk of recurrence (ROR) score that was derived from a trial of early stage ER positive/Her2-negative postmenopausal patients treated with endocrine therapy related to their response to neoadjuvant or adjuvant chemotherapy [22]. Several other gene assays are used or undergoing validation. The latest American Society of Clinical Oncology (ASCO) guideline finds that the strongest level of evidence currently supports the use of either Oncotype DX Recurrence Score or PAM50 ROR [23].

Triple negative breast cancer comprises up to 20% of invasive breast cancers, and in itself it represents a heterogeneous group of subtypes further defined by additional molecular markers. Some of these subtypes are among the most aggressive poor prognosis phenotypes in breast cancer, while others have a relatively good prognosis. New biomarkers for association of triple negative cancers are under investigation. The term “basal-like” is not standardized but generally refers to breast cancers with certain gene expression profiles, including lack of ER, PR, and Her2 expression, expression of basal cytokeratins (CK5/6, 14, or 17), and/or EGFR. These tumors also tend to be high grade and have high mitotic indices, lymphocytic infiltrate, and necrotic or fibrotic areas [24]. While the majority of triple negative cancers are also basal-like, the two categories are not completely synonymous. There is an established link between basal-like and cancers arising in BRCA1 germline mutated carriers. In attempts to better characterize this high risk class of breast cancers, investigators are harnessing “omics” technologies, which have identified at least 6 triple negative subtypes based on gene expression, molecular pathways, and response to therapeutic agents [25]. Such complex diagnostics involve large volumes of data and require validation in clinical trials that are able to evaluate outcomes in such small subgroups of patients. One such novel clinical trial is I-SPY 2 [26], which uses pathologic response to neoadjuvant chemotherapy as the primary endpoint for assessing safety and efficacy of novel agents in stage II-III breast cancer. Results of combining the anti-PD1 antibody, pembrolizumab, with chemotherapy in Her2-negative patients was reported at the ASC0 2017 annual meeting and showed that triple negative patients had the highest pathologic complete response at 40% among all Her2-negative patients, representing an improvement in response when compared to chemotherapy alone [27]. While endpoints such as pathologic response must ultimately translate into overall survival benefit, this novel trial design has allowed more rapid assessment of both molecular data and response to therapy to inform future studies.

Many classes of targeted agents are under investigation in response to actionable mutations being characterized. There are over 70 approved drugs for the treatment of breast cancer, used in many different sequences and combinations [28]. Among these, some of the greatest interests lie in immunotherapy agents. A current approach uses monoclonal antibody blockers of immune inhibitory proteins such as CTLA-4 and PD-1/PD-L1. Several clinical trials in advanced or metastatic breast cancer with immune agents including nivolumab, pembrolizumab, and atezolizumab have been completed and show promise [29], although complex interactions between the tumor immune environment, host immune system, and timing of therapy need extensive further study before routine clinical use. Immune modulators as currently designed are not effective as single agents and are often used in concert with other cytotoxic agents. An intriguing approach in limited, or oligometastatic, disease involves the use of ablative radiotherapy doses to an index lesion in order to activate host immunity through enhanced antigen presentation in combination with immunotherapeutic agents [30].

Although not in routine clinical use currently, next-generation DNA sequencing of tumor tissue can identify cancer related genomic changes in individual patients' tumors. Studies using this technology have identified the most commonly mutated genes in breast cancer, which include PIK3CA, p53, and Her2 amplification mutations in 15% to 30% of breast cancers, although many other candidate genes are present in frequencies <5% [31]. These data provide information for development of targeted agents, including immune pathways. Genomic alterations implicated in several actionable pathways have been identified, including endocrine resistance, Her2 overexpression in apparent Her2-negative cancers and anti-Her2 therapy resistance, and characterization of circulating tumor cell DNA for monitoring treatment response or quantifying residual disease after therapy [32]. Using mutational status to test for response to specific agents has great promise to personalize systemic therapy based on the genomic pathways driving each cancer and to enhance survivorship surveillance.

### 2.3. Radiomics

The emerging field of radiomics involves the use of quantitative features from medical images in prognostic or predictive models as correlated with pathology, genomics, or clinical outcomes. In the breast cancer screening context, such features may be developed to personalize frequency or modality for screening dependent upon more individualized risk factors. A combination of functional imaging tests and molecular subtyping is anticipated to aide in differentiating aggressive or indolent phenotypes. Imaging modalities under investigation include tomosynthesis, contrast enhanced mammography, and MRI. High spatial resolution MRI may assess lesion characteristics to distinguish between DCIS and invasive lesions, potentially mitigating the need for further workup and treatment of the lower risk purely intraductal lesions. MRI can detect biologic features in situ such as cellularity, vascularity, and cell membrane integrity. Quantitative assessment of tissue vascularity can be obtained using dynamic contrast enhancement (DCE), while diffusion weighted MRI imaging (DWI) can measure cell density and membrane disruption [33]. In a prospective study of over 7300 women, breast MRI was performed in addition to screening mammograms [34]. MRI proved to detect a much higher percentage of DCIS and lack of enhancement was associated with lower grade lesions. The cooperative groups Eastern Cooperative Oncology Group (ECOG) and American College of Radiology Imaging Network (ACRIN) are currently accruing to protocol 4112, to study whether breast MRI and the Oncotype DX DCIS score can identify patients with low risk DCIS who may avoid radiation treatment [35].

MRI has been used in several series to characterize invasive cancers and correlate imaging findings with molecular subtype. One group of investigators used data in the National Cancer Institute's (NCI) The Cancer Imaging Archive (TCIA) to evaluate MRI findings with tumor subtype and found that features such as enhancement texture and heterogeneity were significantly correlated with molecular subtype [36]. Other groups found correlation of subtype and kinetic enhancement uptake patterns, suggesting overall that tumor enhancement kinetics are related to biologic characteristics in vivo [37, 38]. The recently activated NRG BR005 cooperative group study is assessing the accuracy of trimodality functional imaging to define the response in the breast and lymph nodes to neoadjuvant chemotherapy and is anticipated to roll into a randomized trial of surgery versus no surgery in women who have imaging defined complete pathologic response [39].

Radiomics may aide in screening, diagnosis, treatment planning for surgery or radiation treatment, evaluating response to therapy, and follow-up care. Radiomic characteristics properly validated might allow avoidance of biopsies or surgery, help define the tissue at risk for radiation target volume delineation, and distinguish posttreatment findings to customize workup and treatment for recurrence.

### 2.4. Surgical Management

In the Halstedian era, the belief was strongly held that more radical surgery was associated with a better outcome or survival rate. Since the seminal trials establishing breast conservation therapy as an equivalent treatment for early stage breast cancer with far less morbidity, the radical surgery concept has been abandoned. The focus of innovation in surgical management over the past two decades has been to reduce the extent of surgery performed [40]. It is already a standard of care to use neoadjuvant systemic therapy to increase the operability of breast cancers and convert women to breast conservation techniques. Once breast conserving surgery was established through several large randomized trials and further validated by the expansive Early Breast Cancer Trialists' Collaborative Group (EBCTCG) series of meta-analyses of these trials [41], the development and validation of sentinel node biopsy were undertaken. This innovation in axillary surgical technique has led to a significant reduction in morbidity with respect to lymphedema risk and arm function, improving quality of life for many breast cancer survivors. Clinicopathologic features such as number of positive nodes as determinants of adjuvant therapies are giving way to intrinsic subtyping and genomic profiling and are becoming much less important for guiding decision making for systemic therapy selection.

Current innovations are designed to further lessen the impact of surgical management, whether by refining the surgical techniques or by omission of surgery in selected cases. Studies have shown that axillary radiation is effective in controlling subclinical disease in clinically node negative patients [42], potentially obviating the need for axillary node biopsy and further reducing the risk of lymphedema. Intrinsic molecular subtype predicts both the risk or distant metastasis as well as the risk of local-regional recurrence after both mastectomy and breast conserving surgery [15]. Using such information in the design of clinical trials may allow refinement of the extent of surgery required as well as the use of radiotherapy and systemic therapies. For example, intrinsic luminal A cancers have an extremely low risk of nodal metastasis, distant metastasis, and local recurrence. With the use subtyping as well as additional predictive and prognostic gene assays, it may be possible to select low risk patients for omission of therapy and reduce overtreatment. An ongoing Patient Centered Outcomes Research Institute (PCORI) grant sponsoring a multi-institutional clinical trial is asking that very question in women with noninvasive ductal carcinoma in situ (DCIS), or stage 0, breast cancer. Known as Comparing Operative to Medical Endocrine Therapy (COMET) for low risk DCIS trial, the study randomizes women with DCIS (grade 1-2, ER or PR positive) to surgery with or without radiation versus active surveillance consisting of mammograms every 6 months for 5 years, and endocrine therapy is allowed on both arms [43].

The use of breast and even axillary surgery will likely be needed for the foreseeable future, particularly for patients with more locally advanced disease. This patient population is the focus of optimizing the combination of surgery, systemic therapy, and radiation therapy. Neoadjuvant systemic therapy is widely used and often leads to downstaging, which challenges decision making regarding optimal postoperative treatment as defined by pathologic response as well as molecular subtype. Multiple innovative approaches in breast reconstruction techniques, including skin and nipple sparing mastectomy, techniques to preserve lymphatics and nerves, and oncoplastic rearrangements in women requiring larger volume resections to achieve breast preservation are enhancing quality of life and cosmetic outcomes for these women [44].

### 2.5. Radiation Therapy

Until recently, radiation for breast cancer was quite uniformly applied with respect to target volumes and dose. Radiotherapy treatment decisions revolved around clinical stage, especially the tumor size and the presence of positive nodes, and other pathologic features such as margin width. Target volumes were generally confined to whole breast or chest wall radiation, without or without nodal volumes, and were usually treated with conventional fractionation schemes that took 5 to 7 weeks to complete. About 15 years ago, alternate treatment approaches began to be reported. Hypofractionated regimens for whole breast radiation shortening overall treatment times from 6 to 7 weeks to 3 to 4 weeks have been well validated in several randomized trials and indeed appear to be as efficacious as well as potentially less toxic than conventional fractionation regimens [45]. Accelerated partial breast irradiation and intraoperative radiotherapy techniques have also been validated in several large randomized trials and multiple multi-institutional series as safe and efficacious for many women with early stage breast cancers, further reducing treatment times to 1 to 5 days [46]. These alternate techniques promise to increase access to breast conservation treatment among women who are socioeconomically or geographically challenged to participate in long courses of daily treatment and to reduce omission of treatment in women who may benefit from radiotherapy after lumpectomy. These techniques also reduce acute and late toxicity. Ongoing studies are defining alternate fractionations in the postmastectomy and node positive settings and after neoadjuvant chemotherapy. Current American Society for Radiation Oncology (ASTRO) [47, 48] and Groupe Européen de Curiethérapie (GEC) and the European Society for Radiotherapy and Oncology (GEC-ESTRO) [49] clinical guidelines encourage the adoption of these alternate techniques in properly selected patient populations to improve outcomes and reduce toxicity.

Clinical trial data are helping to refine personalization of target volumes: whole breast or partial breast; nodal volumes or not; which nodal levels. However, more refinement of target volumes is possible. For example, what is the correct volume of breast tissue to treat for whole breast or partial breast radiation? Exactly which nodal stations are at risk and need to be irradiated? Current techniques use anatomical landmarks such as the CT-defined breast tissue or the surgical bed plus a uniform expansion margin, and clinicopathologic features to define the risk of local-regional recurrence to determine target volumes. A pair of ongoing studies (NSABP B51 [50] and Alliance A011202 [51]) are defining the indications for local-regional radiation treatment and target volumes after neoadjuvant chemotherapy and surgery. Studies of intrinsic molecular subtypes have shown that local-regional recurrence is predicted by subtype [15]. As with distant recurrence risk, luminal A tumors have the lowest risk and triple negative or basal-like have the highest risk of local-regional recurrence. To date, there have been few prospective randomized trials to report local-regional recurrence outcomes based on molecular subtypes or to incorporate subtype into their study design.

There are several areas of interest in personalizing radiotherapy use in breast cancer. One major focus is to reduce overtreatment of low risk patients whose cancers fall below the 10–20% risk of local recurrence that defines a survival benefit for breast radiation [41]. There are several ongoing multi-institutional trials using gene arrays to select patients for observation (omission of radiation) after breast conserving surgery. The IDEA study [52] is using a low risk Oncotype DX Recurrence Score and the Precision study [53] is using a low risk PAM50 ROR for selection of stage I breast cancer patients for omission of radiation after lumpectomy. The premise of both studies is that tumor biology as defined by functional gene assays previously shown to predict local recurrence will be a better methodology for selection of patients for observation than previous unsuccessful trials which used clinicopathologic surrogates of tumor behavior.

Another important area of interest is the use of gene assays of radioresponsiveness to predict which cancers are more or less resistant to radiation. Such studies start to define not only who may benefit from radiation at all, but also what dose of radiation is needed to achieve optimal tumor control probability. The radiosensitivity index (RSI) developed by Torres-Roca and colleagues uses a systems biology approach and fraction of cells surviving 2 Gy (SF2) to define a clinically validated molecular signature that estimates radiosensitivity of multiple tumor types, including breast cancer [54]. In several breast cancer cohorts, RSI predicts for improved relapse free and distant metastasis free survival only in radiosensitive patients who were irradiated. RSI defined radioresistant and sensitive subpopulations especially among triple negative patients and distinguished outcomes by radiation dose in RSI subpopulations [55]. These investigators have proposed a genome-based model for adjusting radiotherapy dose (GARD) based on data from multiple clinical cohorts. The GARD shows a range of values across and within tumor types but generally agrees with clinical observations of known sensitive and resistant tumors and is associated with longer survival in irradiated breast cancer patients with higher (more sensitive) GARD values [56]. The heterogeneity within tumors indicates an opportunity to begin to use such assays to triage radiation dose to individual patients. A different gene assay has been developed by Speers and colleagues using clonogenic survival assays to identify intrinsic radiosensitivity as correlated to gene expression in breast cancer cell lines, generating a radiation sensitivity signature (RSS) [57]. The RSS was cross validated and found to be the most significant predictor of local recurrence as compared to other clinicopathologic features.

The promise of precision medicine for radiation therapy in breast cancer is to develop an array of diagnostic, predictive, and prognostic tests including radiomics, molecular subtyping, gene panels predicting risks of local-regional recurrence, and distant metastases, as well as inherent tumor radioresponsiveness. Such a toolbox will allow for individualized treatment decisions based on likelihood of indolent versus aggressive disease, treatment of the appropriate volume of breast and nodal volumes to eradicate microscopic cells harbored in the highest risk tissues and to deliver the correct dose of radiation based on the individual tumor radiosensitivity.

## 3. Conclusion

Precision medicine holds the promise of truly personalized treatment which provides every individual breast cancer patient with the most appropriate diagnostics and targeted therapies based on the specific cancer's genetic profile as determined by a panel of gene assays and other predictive and prognostic tests. Intense research is being conducted in a wide array of disciplines relevant to breast cancer detection, diagnosis, treatment, and survivorship. This article has provided an overview of some of that research in the arenas of screening and diagnosis, molecular profiling, radiomics, and the major treatment modalities including systemic therapy, surgery, and radiation therapy. As these data emerge and coalesce, the next generation of clinical trials will likely combine panels of molecular assays to drive therapeutic selections. Novel endpoints that allow rapid assessment of these approaches are needed for validation, while traditional endpoints, especially survival and toxicity outcomes, will continue to need to be collected. This is one of the most dynamic periods of basic science and translational research in oncology, with the potential promise to accelerate the ultimate search for the cure for cancer.
